# Overexpression of the *adeB* Efflux Pump Gene in Tigecycline-Resistant *Acinetobacter baumannii* Clinical Isolates and Its Inhibition by (+)Usnic Acid as an Adjuvant

**DOI:** 10.3390/antibiotics10091037

**Published:** 2021-08-25

**Authors:** Nagaraju Bankan, Fathimunnisa Koka, Rajagopalan Vijayaraghavan, Sreekanth Reddy Basireddy, Selvaraj Jayaraman

**Affiliations:** 1 Department of Research and Development, Saveetha Institute of Medical and Technical Sciences, Saveetha Dental College and Hospital, Chennai 602105, India; jai_vijay@hotmail.com; 2 Department of Microbiology, A.C.S.R. Government Medical College and Hospital, Nellore 524004, India; basikanthu@yahoo.co.in; 3 Department of Biochemistry, Saveetha Institute of Medical and Technical Sciences, Saveetha Dental College and Hospital, Chennai 600077, India; jselvaendo@gmail.com

**Keywords:** multidrug resistance, *Acinetobacter* *baumannii*, efflux pumps, *adeB*, (+)Usnic acid

## Abstract

*Acinetobacter* species are among the most life-threatening Gram-negative bacilli, causing hospital-acquired infections, and they are associated with high morbidity and mortality. They show multidrug resistance that acts via various mechanisms. In *Acinetobacter baumannii*, efflux pump-mediated resistance to many antimicrobial compounds, including tigecycline, has been widely reported. Natural compounds have been used for their various pharmacological properties, including anti-efflux pump activity. The present study aimed to evaluate the efflux pump-mediated resistance mechanism of *Acinetobacter* *baumannii* and the effect of (+)Usnic acid as an efflux pump inhibitor with tigecycline. For detecting the efflux pump activity of tigecycline-resistant *Acinetobacter* *baumannii* isolates, microbroth dilution method and real-time quantitative reverse transcription–polymerase chain reaction was used. (+)Usnic acid was added to tigecycline and tested by the checkerboard method to evaluate its efficacy as an efflux pump inhibitor. qRT-PCR analysis was carried out to show the downregulation of the efflux pump in the isolates. Out of 42 tigecycline-resistant *Acinetobacter* *baumannii* isolates, 19 showed efflux pump activity. All 19 strains expressed the *adeB* gene. (+)Usnic acid as an adjuvant showed better efficacy in lowering the minimum inhibitory concentration compared with the conventional efflux pump inhibitor, carbonyl cyanide phenylhydrazone.

## 1. Introduction

Multidrug-resistant *Acinetobacter* species commonly cause hospital-acquired infections. Individuals infected with MDR *A. baumannii* (MDRAb) have an extended period of hospitalization and also have a high mortality rate [1]. According to the Infectious Diseases Society of America (ISDA), *A. baumannii* has been categorized as a “red alert” bacterium that poses a serious threat to antibacterial therapy. Numerous studies have revealed the high prevalence of multidrug resistance in *A. baumannii*, and it can vary significantly by country, city, and hospital. Due to this, MDRAb infections cause high mortality in the critically ill, ranging from 26% to 68% [2]. Among the 18 major drug-resistant threats identified by the Centers for Disease Control and Prevention (CDC), carbapenem-resistant *A. baumannii* requires immediate attention to prevent infection or reduce antibiotic resistance [3]. Numerous hospital and community surveillance programs, as well as the Infectious Diseases Society of America (ISDA), have also identified the “ESKAPE pathogens” (*Enterococcus faecium*, *Staphylococcus aureus*, *Klebsiella pneumonae*, *Acinetobacter baumannii, Pseudomonas aeruginosa*, and *Enterobacter species*), responsible for dangerous nosocomial infections [4,5]. Patients admitted to intensive care units and burn units are the primary carriers of MDRAb due to their immunocompromised state. MDRAb has been associated with a variety of bacterial infections, including sepsis, ventilator-associated pneumonia (VAP), and meningitis. Concerns about *A. baumannii* infections have been raised as a result of multidrug resistance and thus treatment options for of MDRAb are limited [6]. 

Earlier published studies have demonstrated an association between multidrug efflux pumps and the development of resistance to a variety of chemicals, including antibiotics [7,8]. Efflux pumps can contribute to the development of antibiotic resistance by decreasing antibiotic concentrations within the microorganism. Additionally, it has been demonstrated that inhibiting efflux pumps can enhance the antibacterial activity of antibiotics and other drugs [8]. Compounds such as carbonyl cyanide 3 chlorophenylhydrazone (CCCP) and phenylalanine–arginine β naphthylamide (PAβN) have been shown to inhibit a variety of efflux pumps, including AdeABC, AdeFGH, and AdeIJK, although these are not approved for clinical use due to their toxicity [8,9]. 

AdeABC consists of three proteins: an outer membrane protein denoted as *adeC*, a transporter protein denoted as *adeB*, and a fusion membrane protein denoted as *adeA*. The function of *adeB* is to transport substances from the cytoplasm or within the phospholipid bilayer, which is an important mechanism of the development of resistance in *Acinetobacter* species [10]. The expression of AdeABC is regulated by a two-component system called AdeR–AdeS, which consists of a response regulator and a sensor kinase [11]. Increased expression of the resistance–nodulation–cell division (RND) genes AdeIJK, AdeFGH, and AdeABC has been associated with tigecycline resistance [12,13,14]. Overexpression of the *adeB* gene, which encodes the *AdeB* efflux protein, is associated with MDRAb strains [15].

Tigecycline (TGC) was one of the first glycylcyclines approved by the Food and Drug Administration (FDA) because of its potent antimicrobial activity against MDRAb. Tigecycline and colistin are used as alternative treatments for carbapenem-resistant *Acinetobacter* isolates [16]. Antibiotic resistance to tigecycline has been identified in some instances as a result of unrestricted use. Studies have shown that the resistance rates of tigecycline and colistin are 74.2% and 53.1%, respectively [17,18]. Previous studies also demonstrated the role of increased *adeB* gene expression in tigecycline-resistant *Acinetobacter* isolates [19].

The presence of multidrug resistance in these bacteria necessitates the development of novel therapeutic strategies and drugs. Due to their safety, “natural” products derived from plant materials have gained global attention as new antimicrobial compounds, particularly in traditional medicine. It is well-established that the secondary metabolites of plants possess antibacterial properties and the ability to modulate resistance. The adverse effects of conventional antibiotics can be minimized by the use of such compounds. Combination therapy has proven effective in the treatment of Gram-negative bacterial infections and drug-resistant tuberculosis in several studies. Numerous studies have shown that a variety of plant-derived bioactive compounds act synergistically with commonly used antibiotics to enhance their efficacy [20].

Usnic acid (UA) is a lichen-derived secondary metabolite that is abundantly present in *Usnea*, *Ramalina*, and *Cladonia*. Secondary metabolites are produced when cyanobacteria and lichens interact. The UA dextro-isomer derived from these lichens has been investigated for a variety of biological properties, including antibacterial activity. Due to its structural and physicochemical properties, UA exhibits a variety of biological effects: antimicrobial, analgesic, anti-inflammatory, antiviral, and anticancer. The antibacterial properties of lichen extracts and compounds have been studied in detail [21,22,23]. UA’s exact mechanism of action is unknown, but it may be due to the inhibition of calcium ion (Ca2+) influx into the cell [24].

The present study aimed to demonstrate the expression of an *adeB* gene in tigecycline-resistant *A. baumannii* isolates and the use of (+)UA as an adjuvant efflux pump inhibitor (EPI).

## 2. Results

### 2.1. Bacterial Isolates

The study included 100 non-repeated preserved isolates from two different centers. The majority of the isolates (39%) were from endotracheal tubes, followed by pus (24%) and other sources (37%). Preliminary results of antibiotic susceptibility testing by the VITEK method on MDR strains revealed that 42 isolates (42%) were resistant to tigecycline. The antimicrobial susceptibility profiles of 100 isolates for various antibiotics and tigecycline, as determined by VITEK, are shown in Table 1. Except for colistin, all other antimicrobial agents demonstrated a high level of resistance (*p* ˂ 0.001).

### 2.2. MIC Determination of Tigecycline

All 42 tigecycline-resistant isolates identified by VITEK were further assessed by the microbroth dilution method to confirm their tigecycline resistance. The MIC range for tigecycline was found to be 4 µg/mL to 128 µg/mL. As there were no specific breakpoints for tigecycline susceptibility, the US FDA-recommended breakpoint values (Enterobacteriaceae ≤2 mg/L as sensitive, 4 mg/L as intermediate resistance, and ≥8 mg/L as resistant) were considered for testing *Acinetobacter* isolates against tigecycline [25]. When the MIC was determined using the microbroth dilution method in the 42 tested isolates, 40 isolates were found to be resistant to tigecycline (≥8 µg/mL), while two isolates were found to have intermediate resistance to tigecycline (4 µg/mL). The detailed antimicrobial susceptibility profiles of all 42 tigecycline-resistant *A. baumannii* isolates are provided in the supplementary file.

### 2.3. Detection of Efflux Pump Activity Using CCCP as an EPI

Out of 42 tigecycline-resistant isolates, only 19 isolates demonstrated a ≥4-fold reduction in tigecycline resistance when treated with tigecycline and CCCP compared with tigecycline alone, as demonstrated in Table 2.

### 2.4. Quantitative Analysis of the adeB Gene in A. baumannii Isolates

**Figure 1:**
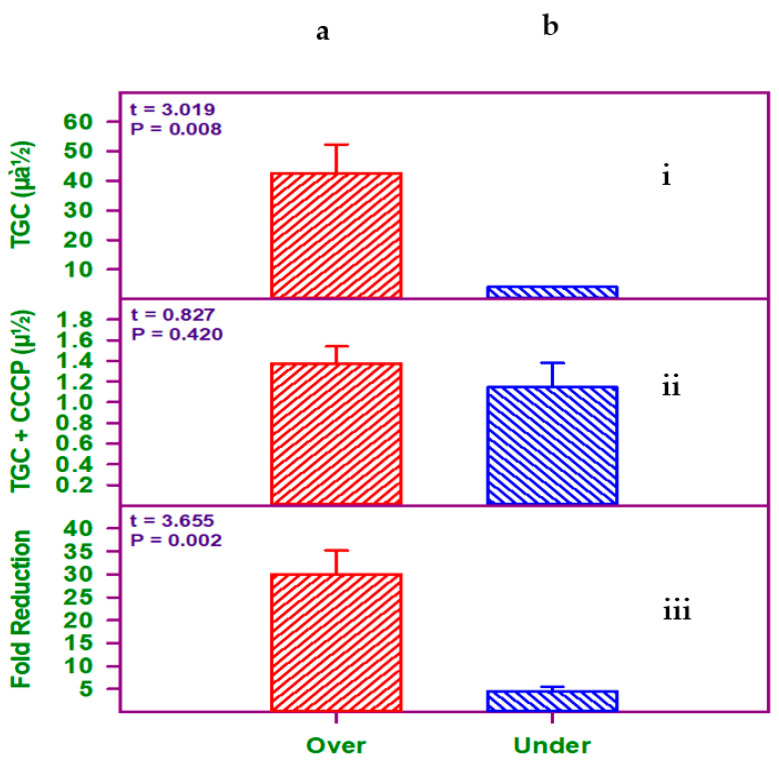
(**i**) (**a**) Resistant isolates compared with (**b**) intermediate resistance isolates, indicating statistical significance (*p* = 0.008) (n = 42 isolates; a = 40 isolates; b = 2 isolates). (**ii**) The fold reduction in tigecycline resistance between the (**a**) resistant isolates and (**b**) the intermediate resistance isolates when treated with CCCP as an EPI. No statistical significance was observed (*p* = 0.420) (n = 19 isolates; a = 12 isolates; b = 7 isolates). (**iii**) There was a statistically significant difference between the mean values of (**a**) high expression and (**b**) low expression of the *adeB* gene according to the qRT-PCR analysis (*p* = 0.002) (n = 19 isolates; a = 12 isolates; b = 7 isolates). All values are means ± SEM.

Real-time quantitative reverse transcription–polymerase chain reaction (qRT-PCR) was used to detect *adeB* gene overexpression in 19 tigecycline-resistant isolates, which showed a ≥4-fold reduction in tigecycline resistance when samples were treated with CCCP (CCCP-positive samples). A tigecycline-sensitive strain was used as a control to compare the expression of the CCCP-positive strains in this analysis. Twelve (63.2%) of the 19 strains had a high level of expression of the *adeB* gene. When treated with CCCP as an EPI, all of these 12 strains had a higher fold reduction in MICs. The remaining seven (36.8%) of the 19 strains showed a lower level of gene expression. The statistical significance of the all three methods has been mentioned in detail in Figure 1.

### 2.5. Antibacterial Activity of (+)Usnic Acid against the AI 7574 Isolate

The isolate AI 574 was chosen for further antibacterial activity analysis via the microbroth dilution method due to its high MIC (128 µg/mL) and its high fold reduction (64-fold) when treated with CCCP. The antibacterial activity of (+)Usnic acid against AI 7574 was determined using the microbroth dilution method in a 96-well microtiter plate. The MIC of (+)Usnic acid was found to be 1024 µg/mL.

### 2.6. Checkerboard Synergy Assay

TGC’s MIC against the AI 7574 isolate was 128 µg /mL, whereas (+)UA showed antibacterial activity at 1024 µg/mL; (+)UA was used at subinhibitory concentrations for this assay. TGC’s MIC was reduced to 0.5 µg/mL (256-fold) when combined with (+)UA, indicating synergy (FICI = 0.03125, FICI 0.5). When CCCP and tigecycline were combined, the MIC of tigecycline was reduced by 1 µg/mL (128-fold), indicating synergy (FICI = 0.0911, FICI ˂ 0.5). These findings indicate that (+)UA has significant synergy with tigecycline. The FICI concentrations are given in Table 3.

### 2.7. Ethidium Bromide Agar Cartwheel Test

**Figure 2:**
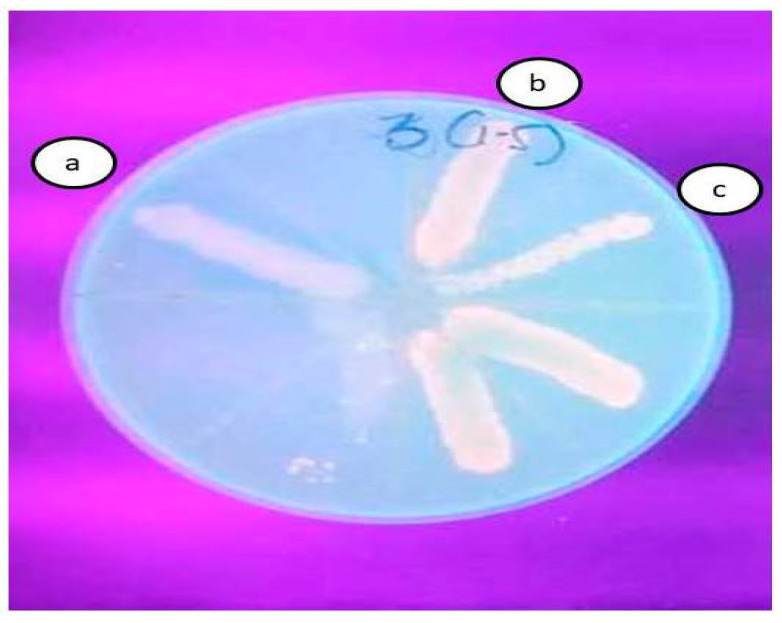
EPI activity of UA, as determined by the ethidium bromide agar cartwheel method, compared with CCCP in AI 7574. (**a**) Untreated; (**b**) treated with (+)UA; (**c**) treated with CCCP. Compared with (**a**) (untreated), fluorescence is seen in (**b**) and (**c**).

The efflux pump inhibitory activity of (+)UA was determined via the EtBr agar cartwheel method using the isolate AI 7574, which expressed the efflux pump *adeB* gene. To determine efflux inhibition, the control (untreated isolate), the standard (treated with 25 µg/mL CCCP), and the test (treated with 32 µg/mL (+)UA) isolates were used in the EtBr agar cartwheel test. In comparison with the control (untreated), the standard and test (treated) isolates both exhibited fluorescence, indicating that efflux pumps were inhibited (Figure 2).

### 2.8. qRT-PCR Analysis of Treated and Untreated AI 7574

**Figure 3:**
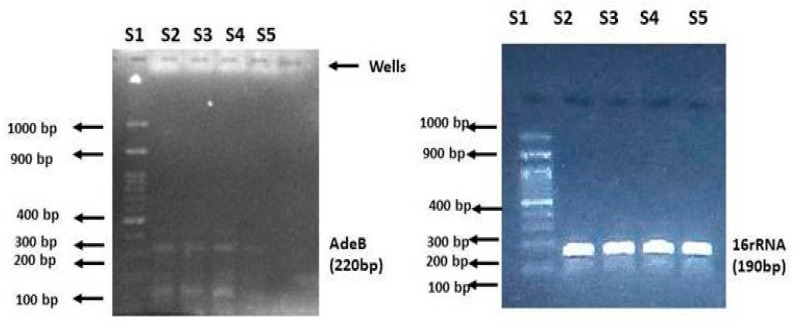
Agarose gel electrophoresis of the treated and untreated AI 7574 isolates. Results of the *adeB* expression analysis for treated and untreated MDRAb isolates. S1: 100 bp ladder; S: untreated isolates; S3: treated isolates + tigecycline; S4: treated isolates + tigecycline + CCCP; S5: treated isolates + tigecycline + (+)UA.

**Figure 4:**
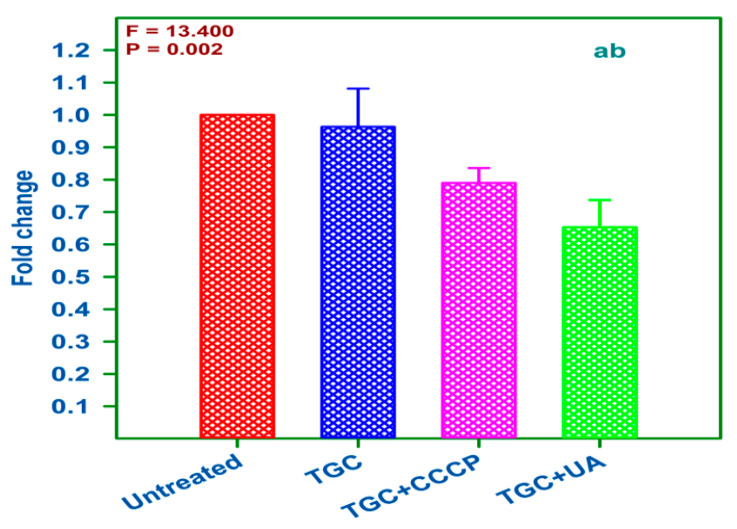
Efflux pump inhibition activity determined by qRT-PCR for AI 7574. Values are presented as means ± SEM (n = 3 each, triplicate data of each group). Untreated: AI 7574 isolate alone; TGC = tigecycline alone; TGC + CCCP = tigecycline + carbonyl cyanide m-chlorophenylhydrazone; TGC + (+)UA = tigecycline + (+)Usnic acid. Compared with the standard, a statistical reduction in expression levels with TGC + (+)UA was seen. *P* is the statistically significant difference in the mean values among the groups. *F* is the cumulative probability distribution among the groups. a: Significantly different from the untreated group. b: Significantly different from the TGC group.

The differences in the *adeB* gene expression levels between untreated and treated strains are illustrated in Figure 3, as determined by agarose gel electrophoresis. The 16Sr RNA gene was used as an internal control to ensure that equal amounts of RNA were used in the qRT-PCR analysis. The results of qRT-PCR between the treated and untreated samples revealed that the expression of the *adeB* gene was significantly reduced in the presence of a combination of TGC and (+)UA (0.65-fold reduction) when compared with the standard group; the combination of TGC and CCCP (0.79-fold), TGC alone (0.963-fold), and the untreated group (1-fold) showed a statistically significant difference among the groups, as shown in Figure 4 (*p* = 0.002). The *adeB* gene was highly expressed before exposure to (+)UA and TGC. The *adeB* gene expression level remained relatively constant in the samples and controls.

## 3. Discussion

The FDA recommends the use of antibiotics such as colistin, minocycline, and tigecycline to treat MDRAb infection [26]. However, tigecycline resistance is becoming more prevalent. In the current study, 42% of the *A. baumannii* isolates were resistant to tigecycline, while 58% of the *A. baumannii* isolates were susceptible to tigecycline, which is consistent with the findings of previous reports of 41.3% resistance [27]. The present findings indicate that in 45.23% of isolates, the MIC was reduced more than fourfold when CCCP was used. In previous studies, CCCP significantly reduced the MIC of *Acinetobacter* species strains two- to fourfold in 51.35% of isolates [28]. The current study found that 12 (64.3%) of the 19 *A. baumannii* isolates were resistant to tigecycline and expressed a high level of *adeB* gene expression, as well as an increased fold reduction in response to tigecycline when combined with CCCP. The remaining seven (36.84%) of the 19 isolates showed a low level of gene expression with a lower fold reduction in response to CCCP, which is consistent with the findings of Yang et al., who found that 50% and 70% of *adeB* and *abeM* genes were overexpressed, respectively. Tigecycline resistance was reduced when tigecycline combined with CCCP as an EPI [29]. The AdeABC efflux pump may play a significant role in the tigecycline resistance of *Acinetobacter* species [29]. The overexpression of *adeB* in the isolates that exhibited the strongest MIC fold reduction when treated with EPI (EPI-responsive isolates) demonstrates the action of efflux pump inhibition. Inhibition of the RND functional drug efflux system, specifically *adeB*, is possible [15]. These findings establish a link between drug efflux pumps and resistance in isolates of *Acinetobacter*. The current study found that all 19 *A. baumannii* (100%) isolates carried the *adeB* gene, which is consistent with previous findings that 100% of *A. baumannii* carried the *adeB* gene [30].

Even though drug efflux pumps play a critical role in the emergence of MDR and extremely drug-resistant (XDR) bacterial strains, currently, there is no clinically approved EPI with which to inhibit these efflux pumps [8]. Efforts are being made to develop a more effective EPI with fewer adverse effects and increased efficacy. Suppression of the RND efflux pump is a promising strategy for overcoming the problems of MDR and XDR. A promising approach is to inhibit RND-type efflux pumps to target their structure, function, and relative expression. This can be accomplished via a variety of approaches, including (a) structurally modifying the existing antibiotics, (b) interfering with the assembly of the pump when protein–protein interfaces are targeted, and (c) inhibition of the proteins found in the inner and outer membranes and inhibition of efflux pump expression [8,31]. (+)UA’s inhibitory activity can be used as an adjuvant with tigecycline against the efflux pump. UA has been studied as an effective efflux pump inhibitor (EPI) against Gram-positive bacteria and *Mycobacterium* species [20,32]. In the present study, the synergistic effect of (+)UA with tigecycline in reducing tigecycline resistance in a highly expressed isolate was found to be very effective when compared with the standard treatment (CCCP).

The current study investigated the effect of (+)UA’s antibacterial activity to determine a subinhibitory concentration for further investigation as an EPI. If the MIC of a secondary metabolite is between 100 and 1000 g/mL, it is considered bactericidal [33]. The MIC of (+)UA was found to be 1024 µg/mL in this study, which is comparable with those of previous studies, in which the MIC was found to be >500 µg/mL; it was also found to have a resistance modulating effect [34]. The combination of (+)UA and TGC resulted in a 256-fold reduction in MIC for the clinical isolate AI 7574. (+)UA was found to be capable of modulating polymyxin resistance against *Pseudomonas aeruginosa* in a similar investigation [34]. Notably, (+)UA was able to restore the activity of the last-line antibiotic tigecycline, to which AI 7574 had developed resistance. The combined FIC index of (+)UA in combination with tigecycline indicated synergism. UA synergism was observed with norfloxacin against MRSA [20], and with clarithromycin against *Mycobacterium abscessus* [32]. According to the FIC index, the synergistic effect of (+)UA and TGC was superior to that of CCCP and TGC. When compared with the efflux active strain AI 7574, fluorescence was detected for the (+)UA- and CCCP-treated strains using the ethidium bromide agar cartwheel method. This finding was consistent with an earlier study in which UA was found to be more effective than CCCP against the clarithromycin-resistant *Mycobacterium abscessus* strain AT 52 [32].

Several strategies for inhibiting efflux pump activity have been developed, including repression of the efflux pump gene, disruption of the pump assembly, blocking the outer membrane channels (OprM), altering the chemical structure of antibiotics, and inhibiting efflux binding sites [8]. The qRT-PCR findings support previous reports that (+)UA can be used as an EPI to restore antibiotic action [20,32]. The qRT-PCR approach has the advantage of being rapid and sensitive, as well as adaptable in clinical laboratory settings [35]. 

The inhibitory activity of (+)UA in combination with tigecycline on efflux pumps may be mediated by the following mechanism: UA has been shown to inhibit calcium ion (Ca2+) influx into the cells and destabilize the bacterial membrane [24,34]. These findings support the beneficial role of (+)UA as a MDRAb EPI. By reducing antibiotic resistance, (+)UA can act as an adjuvant for Gram-negative bacterial efflux pump inhibition. Additionally, it may be a better EPI for isolates with a low level of *adeB* gene expression. UA has been found to be very safe in animal models [36].

In this study, we demonstrated the EPI activity of (+)UA by using the EtBr agar cartwheel method and by testing for reduced *adeB* gene expression using qRT-PCR, which resulted in a decrease in TGC resistance in *A. baumannii*. Recent studies have revealed that usnic acid exerts its EPI activity by dissipating membrane potential or by competing with antimicrobial substrates for the same regions in the efflux pump [20,32]. This may be one of the mechanisms by which usnic acid inhibits the efflux pump and restores the efficacy of antibiotics or tigecycline when used in combination with (+)UA. This mechanism is comparable with that of carbonyl cyanide m-chlorophenyl hydrazone (CCCP), a protonophore that induces dissipation of the membrane potential, thus affecting all secondary transport-dependent effluxes; CCCP has been demonstrated to be an RND pump inhibitor [37].

## 4. Experimental Methods

### 4.1. Sources of Bacterial Isolates

MDRAb isolates (n = 100) (preserved at −70 °C in trypticase soy broth supplemented with 30% glycerol) collected between 2017 to 2019 from two different states in India (SVS Medical College and Hospital, Mahabubnagar Telangana, and ACSR Medical College and Hospital, Nellore, Andhra Pradesh). Bacterial isolates were identified using the VITEK 2 system (bioMérieux, Marcy l’Etoile, France). Molecular studies were performed at the Saveetha Institute of Medical and Technical Sciences (Chennai, India). Isolates were obtained from cerebrospinal fluid (CSF), pus, sputum, endotracheal tube, blood, wound swabs, urine, and pleural fluid.

### 4.2. Chemicals and Reagents

Mueller–Hinton agar and Luria–Bertani broth were obtained from Himedia (Mumbai, India), tigecycline antibiotic powder was obtained from Cipla Ltd., (Mumbai, India), and CCCP (C2759) and ethidium bromide were obtained from Sigma Aldrich, St. Louis, MO, USA).

### 4.3. Determination of Tigecycline Resistance

Tigecycline resistance was determined using the microbroth dilution method in Mueller–Hinton broth. The susceptibility testing results were interpreted following the US FDA’s guidelines for tigecycline sensitivity [38].

### 4.4. Detection of Efflux Pump Activity Using CCCP as an EPI

The MIC of tigecycline at concentrations ranging from 0.5 to 256 µg/mL was determined using the microbroth dilution technique in the presence and absence of CCCP at a final concentration of 25 µg/mL [28].

### 4.5. Total RNA, cDNA Synthesis, and Real-Time PCR

Fresh LB broth was used for the subculture. Bacteria were cultured overnight at 37 °C to the mid-log phase (OD at 600 nm) and then used for total RNA isolation, using a total RNA isolation reagent (TRIR) (Ab gene house, Surrey, United Kingdom). Next, 2 µg of RNA was transcribed using the reverse transcriptase RT kit from Eurogentec (Seraing, Belgium), and 16 s rRNA was used as a housekeeping gene. Genes were amplified using the SYBR green master mix (Takara, Japan) in a qRT-PCR system (CFX96 Touch Real-Time PCR Detection System (Bio-Rad, Hercules, CA, USA). The PCR was performed as initial denaturation at 95 °C for 5 min, followed by 40 cycles of 95 °C for 30 s, 59–60 °C for 30 s, and 72 °C for 30 s. Relative quantification was calculated from an analysis of the melt and amplification curves. The relative expression was determined using the 2-ΔΔCT method, and the RNA input was normalized to the housekeeping gene 16 s rRNA. The expression level of each gene was calculated using the fold change relative to the transcriptional level of the corresponding gene in the *A. baumannii* tigecycline-sensitive strain (negative control). All experiments were repeated three times [39]. The sequences of the primers used in this study were taken from previous reports [40,41].

### 4.6. Antibacterial Activity of (+)Usnic Acid against AI 7574 A. baumannii

The MIC of (+)UA against AI 7574 *A. baumannii* was determined using a microbroth dilution assay in 96-well microtiter plates [41]. For this, 100 µL of bacterial inoculum at a 10^6^ CFU/mL concentration and supplemented with Mueller–Hinton broth was added to wells, followed by 50 µL of serial dilutions of (+)UA ranging from 1 to 1024 µg/mL. The plates were then incubated at 37 °C for 24 h. The MIC values were interpreted according to CLSI guidelines [34].

### 4.7. Checkerboard Assay

The checkerboard experiment was used to determine the appropriate concentration of (+)UA in the presence of tigecycline for the antibiotic potentiation assay. The concentrations of (+)UA were determined to be subinhibitory. Mueller–Hinton broth was added to each well of the microtiter plate, with increasing concentrations of tigecycline in the rows and of (+)UA in the columns. The wells were then inoculated with a bacterial inoculum containing 10^6^ CFU/mL and incubated overnight at 37 °C. The MIC was calculated based on non-turbid or minimal growth in the wells in each column and row of the microtiter plate (no growth/lowest absorbance). The fractional inhibitory concentration index (FICI) was determined using the formula with MIC values of the combinations. The following interpretive criteria were applied: according to the odds study, synergy exists when the FICI value is less than 0.5; indifference exists when the FICI value is greater than 0.5, and antagonism exists when the FICI value is greater than 4 [42]. The fractional inhibitory concentration index was calculated as:

∑ FIC = FICA + FICB (FIC_A_ = MIC of C_A_ in combination/MIC of C_A_, FIC_B_ = MIC of C_B_ in combination/MIC of C_B_,

where A and B are the concentrations of tigecycline and (+)UA, respectively.

### 4.8. Ethidium Bromide Agar Cartwheel Test

The efflux pump inhibitory activity of (+)UA was determined using an ethidium bromide cartwheel assay (modified from the method of Arya et al.). A clinical isolate of AI 7574 with an active efflux pump (10^6^ CFU/mL) in MHB supplemented with 32 g/mL (+)UA dissolved in DMSO was cultured overnight. The overnight culture (10^8^ CFU/mL) was then diluted to 10^6^ CFU/mL and treated for 30 min with the same concentration of ((+)UA in MHB; the same procedure was performed for the standard using 25 g/mL CCCP dissolved in DMSO and for the untreated culture. After 30 min of incubation, the cultures were streaked onto freshly prepared EtBr MHA plates. The plates were incubated overnight at 37 °C and examined for fluorescence under ultraviolet light [42].

### 4.9. Expression Analysis of AI 7574 When Treated with (+)UA and Tigecycline

The expression of the *adeB* gene was determined by qRT-PCR as described previously. The AI 7574 isolates (untreated, incubated with TGC alone, 25 µg/mL CCCP + TGC, or 32 µg/mL (+)UA + TGC) were grown overnight. After overnight incubation, the OD of the suspension was adjusted to 0.5 McFarland standard. RNA isolation and cDNA were synthesized; then, changes in the *adeB* gene expression level were determined for the isolate AI 7574 by using the real-time PCR method, as described in Section 4.5, in the following groups: (i) untreated isolates, (ii) TGC alone, (iii) TGC + CCCP, and (iv) TGC + (+)UA [39,42].

### 4.10. Statistical Analysis

The antimicrobial susceptibility was expressed as a frequency and analyzed using the χ^2^ test. The other data were expressed as means ± SEM. The expression studies were analyzed by Student’s *t*-test. The efflux pump inhibition activity was analyzed by one-way ANOVA with Bonferroni’s *t*-test. A probability of 0.05 or less was considered to be statistically significant. The data analysis and graph plotting were carried out in SigmaPlot 14.5 (Systat Software, San Jose, CA, United States of America). 

## 5. Conclusions

The present study showed the overexpression of the *adeB* gene in clinical isolates of *A. baumannii* and a reduction in tigecycline resistance of more than fourfold with CCCP. The RND-type efflux pumps are promising targets for inhibition. (+)Usnic acid can be used as an adjuvant as an efflux pump inhibitor in MDRAb. Plant-derived compounds can act as adjuvants to antibiotics to increase their ability to treat infectious diseases.

## Figures and Tables

**Table 1:** Antimicrobial susceptibility profile of *Acinetobacter* isolates (n = 100).

Sl. No:	Antibiotic	Resistance	Sensitive
1	Piperacillin/tazobactum	53	47
2	Amikacin	49	51
3	Ciprofloxacin	56	44
4	Tetracycline	54	46
5	Imipenem	47	53
6	Meropenem	50	50
7	Tigecycline	42	58
8	Colistin	2	98

Χ^2^ = 87.26, df = 7, *p* ˂ 0.001 (statistics are expressed as the frequency of the antimicrobial susceptibility profile in 100 *A. baumannii* isolates). Statistical analysis was performed to detect the susceptibility pattern of the antibiotics used for treating *A. baumannii* and was statistically significant.

**Table 2:** Phenotypic detection of an efflux pump in tigecycline-resistant isolates by using CCCP and the associated MIC fold reduction, along with *adeB* gene expression levels.

Sl.No.	Isolate	TGC Alone (µg/mL)	TGC + CCCP (µg/mL)	Fold Reduction	Expression of *adeB*
1	**AI 1444**	**32**	**1**	**32**	**0.96**
2	**AI 7574**	**128**	**2**	**64**	**1.03**
3	**AI 8164**	**32**	**1**	**32**	**0.97**
4	**AI 646-2**	**64**	**1**	**64**	**0.99**
5	AI 2796	16	16	1	
6	AI 670	8	8	1	
7	AI 7819	64	64	1	
8	**AI 646-5**	**16**	**2**	**8**	**0.3**
9	AI 4185	8	8	1	
10	**AI 829**	**8**	**2**	**4**	**0.2**
11	**AI 6142**	**64**	**2**	**32**	**0.97**
12	AI 1187	16	16	1	
13	AI 2563	64	64	1	
14	AI 6044	8	8	1	
15	AI 7783	16	16	1	
16	**AI 6553**	**8**	**2**	**4**	**0.3**
17	**AI 5678**	**8**	**1**	**8**	**0.3**
18	**AI 6538**	**16**	**2**	**8**	**0.57**
19	AI 8625	32	32	1	
20	**AI 3990**	**32**	**2**	**16**	**0.81**
21	AI 3699	64	64	1	
22	AI 4727	128	128	1	
23	**AI 4888**	**8**	**2**	**4**	**0.4**
24	**AI 3074**	**32**	**2**	**16**	**0.63**
25	AI 3927	4	4	1	
26	AI 6960	128	128	1	
27	**AI 899**	**32**	**1**	**32**	**0.97**
28	**AI 6428**	**16**	**2**	**8**	**0.47**
29	AI 7703	16	16	1	
30	AI 3636	64	64	1	
31	AI 6372	8	8	1	
32	AI 306	32	32	1	
33	**AI 2760**	**64**	**1**	**64**	**0.99**
34	AI 1259	16	16	1	
35	AI 8426	8	8	1	
36	AI 5096	64	64	1	
37	**AI 2540**	**16**	**2**	**8**	**0.43**
38	**AI 7496**	**16**	**4**	**4**	**0.36**
39	**AI 5289**	**64**	**2**	**32**	**0.98**
40	AI 3840	32	32	1	
41	AI 2368	8	8	1	
42	AI 2218	4	4	1	

Fold reduction in tigecycline following treatment with CCCP in tigecycline-resistant isolates. The bold font indicates isolates that showed a ≥4-fold reduction with CCCP; *adeB* expression was analyzed in these isolates. The *adeB* gene expression is given as the fold change in expression levels obtained via qRT-PCR analysis. The empty wells were not analyzed for qRT-PCR as they did not show any fold reduction with CCCP.

**Table 3:** FICI concentrations for TGC + (+)UA and TGC + CCCP.

	Drug A	Drug B	Drug A Concentration in Combination	FIC_A_	FIC_B_	FIC_i_ (FIC_A_ + FIC_B_)
Drug A (TGC) + Drug B (UA)	128	1024	0.666	0.005	0.0260	0.031
Drug A (TGC) + Drug B (CCCP)	128	256	1	0.00781	0.0976	0.105

The test was performed in triplicate and mean values were used for the calculation.

## Data Availability

Data sharing is not applicable.

## Supplementary Materials

The following are available online at https://www.mdpi.com/article/10.3390/antibiotics10091037/s1, Table S1: Antimicrobial Susceptibility profile of 42 tigecycline resistant *A. baumannii* isolates, Figure S1a: Melt curve of *adeB* gene of qRT-PCR analysis for (+)UA + TGC treated and untreated groups Figure S1b: Amplification of *adeB* gene of qRT-PCR analysis for (+)UA + TGC treated and untreated groups.
