# Supplementary material for: Overexpression of the adeB Efflux Pump Gene in Tigecycline-Resistant Acinetobacter baumannii Clinical Isolates and Its Inhibition by (+)Usnic Acid as an Adjuvant
Source: Antibiotics (Basel). 2021 Aug 25;10(9):1037. doi: 10.3390/antibiotics10091037 (PMC8472003; doi:10.3390/antibiotics10091037)
Supplement: Supplementary file 1 [file antibiotics-10-01037-s001.zip › antibiotics-1330654-supplementary.pdf]

Table S1 Antimicrobial Susceptibility profile of 42 tigecycline resistant *A. baumannii* isolates

Isolate	PIT	AK	CIP	TR	IMP	MRP	TGC	CL
**AI 1444**	R	R	R	R	R	R	R	S
**AI 7574**	R	R	R	R	R	R	R	R
**AI 8164**	R	R	R	R	R	R	R	S
**AI 646-2**	R	R	R	R	R	R	R	S
AI 2796	R	R	R	R	R	R	R	S
AI 670	R	R	R	R	R	R	R	S
AI 7819	R	R	R	R	R	S	R	S
**AI 646-5**	R	R	R	R	R	R	R	S
AI 4185	R	R	R	R	R	R	R	S
**AI 829**	R	R	R	R	R	R	R	S
**AI 6142**	R	R	R	R	R	R	R	S
AI 1187	R	R	R	R	S	S	R	S
AI 2563	R	R	R	R	R	R	R	S
AI 6044	R	R	R	R	R	R	R	S
AI 7783	R	R	R	R	R	R	R	S
**AI 6553**	R	R	R	R	R	R	R	S
**AI 5678**	R	R	R	R	R	R	R	S
**AI 6538**	R	R	R	R	R	R	R	S
AI 8625	R	R	R	R	R	R	R	S
**AI 3990**	R	R	R	R	R	R	R	S
AI 3699	R	R	R	R	R	R	R	S
AI 4727	R	R	R	R	R	R	R	R
**AI 4888**	R	R	R	R	R	R	R	S
**AI 3074**	R	R	R	R	R	R	R	S
AI 3927	R	R	R	R	R	R	R	S
AI 6960	R	R	R	R	R	S	R	S
**AI 899**	R	R	R	R	R	R	R	S
**AI 6428**	R	R	R	R	R	R	R	S
AI 7703	R	R	R	R	S	R	R	S
AI 3636	R	R	R	R	R	R	R	S
AI 6372	R	R	R	R	R	R	R	S
AI 306	R	R	R	R	R	R	R	S
**AI 2760**	R	R	R	R	R	R	R	S
AI 1259	R	R	R	R	R	R	R	S

AI 8426	R	R	R	R	R	R	R	S
AI 5096	R	R	R	R	R	R	R	S
**AI 2540**	R	R	R	R	R	R	R	S
**AI 7496**	R	R	R	R	R	R	R	S
**AI 5289**	R	R	R	R	R	R	R	S
AI 3840	R	R	R	R	R	R	R	S
AI 2368	R	R	R	R	R	R	R	S
AI 2218	R	R	R	R	R	R	R	S

(PIT – Piperacillin/ Tazobactum, AK – Amikacin, CIP – Ciprofloxacin, TR – Tetracycline, TGC – Tigecycline, IMP – Imipenem, MRP – Meropenem and CL – Colistin)

(R – Resistant, S – Sensitive)

<u>Figure S1a Melt curve of *adeB* gene of qRT-PCR analysis for (+)-UA + TGC treated and untreated groups</u>

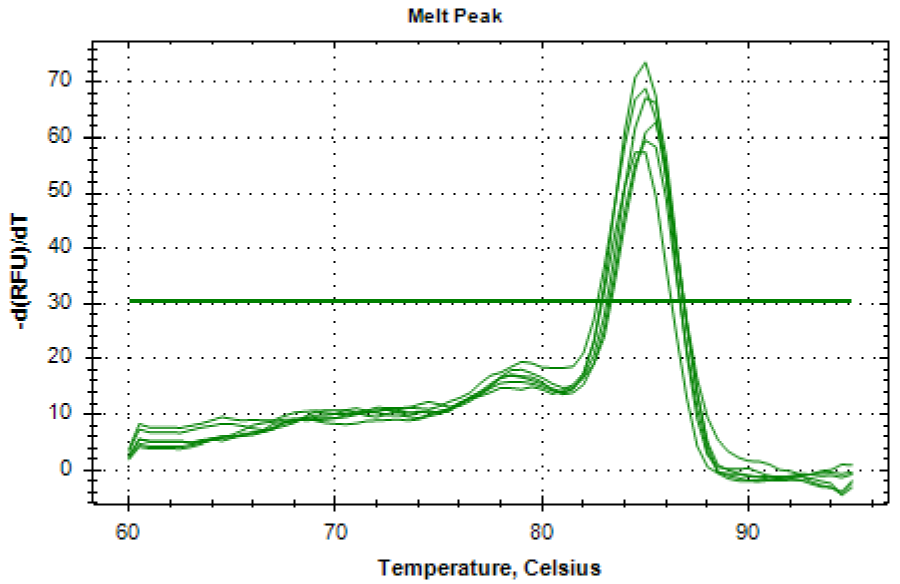

Melt curve analysis for *adeB* gene of treated and untreated groups

Figure S1b Amplification of *adeB* gene of qRT-PCR analysis for (+)-UA + TGC treated and untreated groups

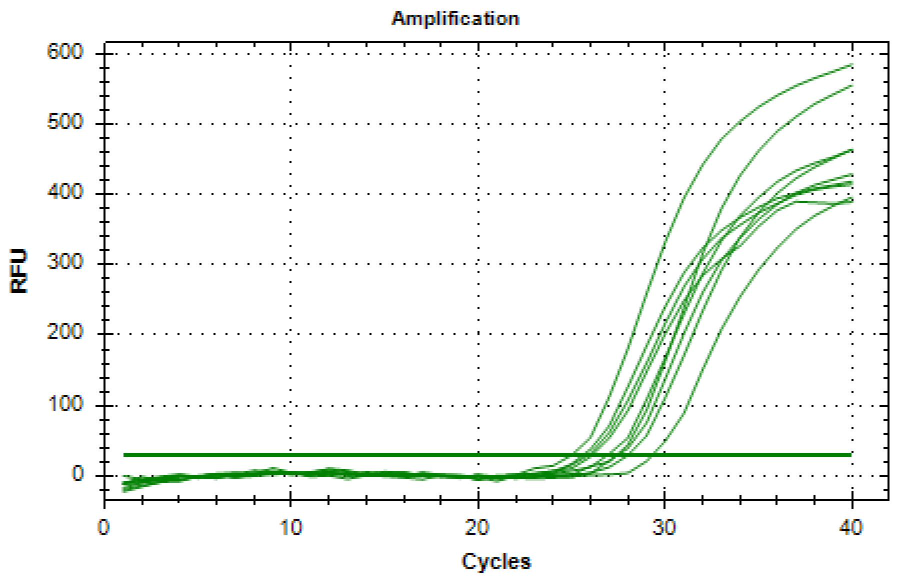

Amplification plot for *adeB* gene of treated and untreated groups
